# HOXB7 drives bladder cancer progression via H-Ras/ERK signaling: a potential therapeutic target and prognostic biomarker

**DOI:** 10.3389/fonc.2025.1645803

**Published:** 2025-09-17

**Authors:** Xulong Chen, Kehua Jiang, Kun Chen, Yu An, Qing Wang, Xiaolong Chen, Peng Zhang, Quliang Zhong, Fa Sun

**Affiliations:** ^1^ School of Medicine, Guizhou University, Guiyang, China; ^2^ Department of Urology, Affiliated Hospital of Guizhou Medical University, Guiyang, China; ^3^ Department of Urology, Guizhou Provincial People’s Hospital, Guiyang, China; ^4^ Center of Prenatal Diagnosis, Guizhou Provincial People’s Hospital, Guiyang, China; ^5^ Central Laboratory, Guizhou Provincial People’s Hospital, Guiyang, China

**Keywords:** bladder cancer, HOXB7, epithelial-mesenchymal transition, MEK/ERK signaling pathway, tumor progression

## Abstract

**Background:**

Bladder cancer (BC) is a common malignancy characterized by high recurrence and poor prognosis. HOXB7, a member of the HOX gene family, is aberrantly expressed in various tumors, but its role in BC remains unclear.

**Methods:**

HOXB7 expression in BC was analyzed using public databases (GEPIA, UALCAN) and validated by immunohistochemistry on a tissue microarray of 36 BC patients. *In vitro* experiments using BC cell lines (5637 and T24) were conducted to investigate the effects of HOXB7 knockdown or overexpression on cell proliferation, apoptosis, migration, invasion, and epithelial–mesenchymal transition (EMT). Western blotting and rescue assays with ERK pathway modulators (Ro67–7476 and PD98059) were performed to assess the involvement of the H-Ras/Raf-1/MEK/ERK signaling cascade. Xenograft mouse models were employed to evaluate tumorigenicity *in vivo*.

**Results:**

HOXB7 was significantly upregulated in BC tissues and cell lines, correlating with advanced tumor stage and poor overall survival. HOXB7 silencing inhibited BC cell proliferation, migration, invasion, and EMT, while promoting apoptosis. Conversely, HOXB7 overexpression produced the opposite effects. Mechanistically, HOXB7 activated the H-Ras/Raf-1/MEK/ERK pathway, as indicated by increased phosphorylation of MEK and ERK. These effects were reversed by pharmacological inhibition or activation of ERK signaling. *In vivo*, HOXB7 knockdown suppressed tumor growth and ERK pathway activation.

**Conclusion:**

This study provides the first comprehensive experimental evidence that HOXB7 drives BC progression via activation of the H-Ras/Raf-1/MEK/ERK pathway. These findings highlight HOXB7 as a potential prognostic biomarker and therapeutic target in BC. Furthermore, our results lay the foundation for future investigations into the broader molecular and immunological networks modulated by HOXB7 in BC.

## Introduction

Bladder cancer (BC) is one of the most frequently diagnosed malignancies of the urogenital system, ranking as the 9^th^ most common cancer worldwide (1). In 2022, there were an estimated 613,791 new cases and 220,349 cancer-related deaths globally (1). Approximately 75% of newly diagnosed BC cases are non-muscle invasive BC (NMIBC). The standard treatment for NMIBC is transurethral resection of the bladder tumor (TURBT), a procedure performed via the urethra to remove visible bladder lesions and obtain pathological specimens for diagnosis. Although TURBT plays a crucial role in early-stage BC management and reduces the risk of progression, NMIBC exhibits a high recurrence rate, with up to 80% of cases recuring within one year, and approximately25% progressing to muscle-invasive BC (MIBC) (2), which carries a high risk of lymph node or distant metastasis (3). Recurrence and metastasis are the main contributors to the poor prognosis and low 5-year survival rate of BC (4). Current targeted therapies for BC remain limited (5), and it is imperative to investigate novel targets for molecular therapy and to obtain a deeper understanding of molecular mechanisms at play to improve treatment outcomes and thus prognosis.

The aberrant expression of HOX genes is associated with the pathogenesis and progression of various cancers (6, 7). Among the HOX gene family, Homeobox B7 (HOXB7) has been identified as a key player in multiple types of cancer, including genitourinary tumors. For example, in prostate cancer (PCa), HOXB7 expression is significantly higher in tumor tissue compared with normal tissue and is strongly correlated with the Gleason score and Tumor Node Metastasis (TNM) stage (8). The upregulation of HOXB7 has been shown to promote the proliferation and migration of PCa cells, especially in those overexpressing miR-384, thereby counteracting the tumor-suppressive effect of miR-384 (9). However, its precise role in BC remains unclear. Given this knowledge gap, it is essential to experimentally determine the expression levels of HOXB7 and to elucidate the molecular mechanisms by which it may influence the progression of BC.

The H-Ras/Raf-1/MEK/ERK signaling pathway is essential for cell growth, differentiation, and survival (10). In cancer, its dysregulation—whether through mutations or oncogenic activation—leads to uncontrolled proliferation and tumor progression (11, 12). When H-Ras, a small GTPase, is activated, it transmits signals to Raf-1, a serine/threonine kinase. Raf-1 subsequently activates mitogen-activated protein kinases (MAPK)/extracellular signal-regulated kinase (ERK) kinase (MEK), which in turn phosphorylates and activates ERK. This cascade of events triggers the activation of various transcription factors that promote cell proliferation and inhibit apoptosis. In cancer, mutations in H-Ras or other pathway components can result in constitutive activation, driving uncontrolled cell growth and tumor formation. Moreover, this MAPK/MEK/ERK pathway can be hijacked by oncogenic signals, further promoting cancer progression (13).

The present study identified HOXB7 as a key regulator of BC progression, with its effects mediated through regulation of the H-Ras/Raf-1//MEK/ERK pathway. These findings establish HOXB7 as both a prognostic biomarker and a potential therapeutic target, paving the way for more precise treatments in BC management.

## Materials and methods

### Human specimens

Paired cancerous and adjacent normal tissue (ANT) samples from 36 patients with bladder urothelial carcinoma were obtained from a commercially available paraffin-embedded tissue microarray provided by Shanghai Weiao Biotechnology Co., Ltd. The inclusion criteria were as follows: (i) histopathological confirmation of bladder urothelial carcinoma; (ii) availability of matched tumor and ANT samples; (iii) no history of neoadjuvant chemotherapy or radiotherapy to minimize the risk of treatment-related molecular alterations; and (iv) complete clinical data, including age, sex, tumor stage, pathological grade, and survival information. Patients with other malignancies or incomplete clinical records were excluded. Based on HOXB7 expression levels, the patients were classified into a high-expression group (HOXB7-high) and a low-expression group (HOXB7-low). Kaplan–Meier survival analysis was performed to compare the overall survival between the two groups, and differences were assessed using the log-rank test. A p-value < 0.05 was considered statistically significant. The 95% confidence intervals were shown by shaded areas in the survival curves. All statistical analyses and visualizations were carried out using R software (version 4.1.3) with the “survival” and “survminer” packages.

### Immunohistochemistry

The paraffin sections were baked at 59˚C for 60 min, deparaffinized in xylene (three times, 10 min each), and dehydrated in anhydrous ethanol (three times, 10 min each). After immersion in distilled water and rinsing with running water for 10–15 min, slides were incubated in 3% H_2_O_2_+NaN_3_ for 10 min to block endogenous peroxidase activity, followed by a 10-min rinse. Antigen retrieval was performed using a microwave (medium-high power) in 1x sodium citrate antigen retrieval solution, with an initial preheating step (1–2 min), followed by 10 min of heating. The slides were then allowed to cool to room temperature and reheated for 12 min under the same conditions. After retrieval, slides were washed twice in PBS (15 min each). Tissue sections were incubated with 5% BSA blocking solution at room temperature for 35 min before primary antibody incubation. Primary antibody incubation was performed overnight at 4°C without prior washing. After four PBS washes (15 min each), slides were incubated with the secondary antibody at 37°C for 35 min, followed by four additional PBS washes (15 min each). Manual hematoxylin counterstaining was performed for 2 min, followed by washing under running water for 10 min. After counterstaining, slides were sequentially washed in PBS (three times, 5 min each). Finally, slides were sequentially dehydrated in ethanol and xylene, mounted with a coverslip, and observed under the microscope (IX73P2F, Olympus Corporation).

### Databases

Gene Expression Profile Interactive Analysis (GEPIA; http://gepia.cancer-pku.cn/) was used to compare the mRNA expression levels of HOXB7 in patients with BC from TCGA/Gene Tissue Expression (GTEx) using a |Log_2_FC| cutoff of 1 and a P-value cutoff of 0.01. The FPKM-normalized mRNA expression data for 414 BC tissues and 19 adjacent non-tumor tissues were obtained from the TCGA-BLCA cohort. For differential expression analysis, the expression values were log2-transformed as log2(FPKM+1) and analyzed using the “limma” package and Wilcox-test in R. The differentially expressed genes (DEGs) were identified with a threshold of |log2FC|≥1 and adjusted P-value <0.05. The UALCAN database (http://ualcan.path.uab.edu/) was used to assess HOXB7 expression levels in tumor tissue compared with normal samples across different tumor subgroups.

*Bioinformatics analysis of the DEGs and signaling pathway.* The R packages “limma” and “ggpubr” were used to identify the DEGs associated with HOXB7 expression between BC and normal samples from the TCGA dataset. The screening criteria included a |log_2_FC| ≥1 and an adjusted P-value of <0.05. The R package “pheatmap” was used to visualize the DEGs. To explore the biological pathways associated with HOXB7 expression, Kyoto Encyclopedia of Genes and Genomes (KEGG) pathway enrichment analysis was performed based on the DEGs using the ‘clusterProfiler’, ‘org.Hs.eg.db’, ‘enrichplot’, and ‘ggplot2’ packages, with a statistical threshold of *P* < 0.05. Gene annotation was based on the ‘org.Hs.eg.db’ database. DEGs between the high- and low-HOXB7 expression groups were identified based on a threshold of |log_2_FC| ≥1 and adjusted P-value <0.05. Enrichment analysis was performed using the enrichKEGG function. *P* < 0.05 was used as the initial cutoff for identifying potentially enriched pathways. The Benjamini-Hochberg (BH) method was used, and pathways with Q-values [false discovery rate (FDR)-adjusted P-values) <0.05 were considered significantly enriched. Q-values reflect the estimated FDR and help ensure the robustness and biological relevance of the identified pathways.

### Cell culture

The human BC cell lines 5637 (CL-0002) and T24 (CL-0227) and the urothelial cell line SV-HUC-1 (CL-0222) were obtained from Procell Life Science & Technology Co., Ltd (Wuhan, China). The human BC cell lines TCCSUP (cat. no. CBP60752) and J82 (cat. no. CBP60311) were purchased from Nanjing Cobioer Biosciences Co., Ltd. The T24, 5637, and J82 cells were cultured in McCoy’s 5A medium (Biological Industries), RPMI 1640 medium, and MEM, respectively, each supplemented with 10% FBS (Gibco; Thermo Fisher Scientific, Inc.). The TCCSUP cells were cultured in MEM supplemented with 10% FBS, 1% non-essential amino acids, and 1 mM sodium pyruvate. The SV-HUC-1 cells were cultured in Ham’s F-12K medium with 10% FBS. All cells were maintained in a humidified incubator at 37°C with 5% CO_2_ and passaged every 2–3 days based on their growth. All experiments were conducted using cells at passages 3–8 to ensure consistency and minimize genetic drift. For loss-of-function and gain-of-function experiments, 1 μM Ro67-7476 (phospho-ERK1/2 activator, MedChemExpress) was added to HOXB7-knockdown 5637 cells, while 10 μM PD98059 (p-ERK1/2 inhibitor, MedChemExpress) was applied to HOXB7-overexpressing T24 cells. All treatments were incubated under identical conditions for 24 h. After incubation, the medium was replaced with fresh media without drugs.

### Transient transfection experiments

The siRNA sequences targeting HOXB7 mRNA and si-NC as negative control were synthesized by Shanghai GenePharma Co., Ltd. The sequences were as follows: si-HOXB7#1 forward, 5’-GAGAGUAACUUCCGGAUCUTT-3’ and reverse, 5’−AGAUCCGGAAGUUACUCUCTT−3’; si-HOXB7#2 forward, 5’-CGGAAAGACAGAUCAAGAUTT-3’ and reverse, 5’−AUCUUGAUCUGUCUUUCCGTT− 3’; si-HOXB7#3 forward, 5’-CGAGAGTAACTTCCGGATCTA-3’ and si-NC forward, 5’-UUCUCCGAACGUGUCACGUTT-3’ and reverse, 5’-ACGUGACACGUUCGGAGAATT-3’.

si-HOXB7 and si-NC were transfected into 5637 cells using Lipofectamine 3000 (cat. no. L3000-015, Thermo Fisher Scientific, Inc.) according to the manufacturer’s protocol. After a 4-h transfection period, the cells were cultured in normal medium for 48 h. Subsequently, the cells were harvested for further experiments. The siRNA achieving the highest knockdown efficiency was selected for subsequent functional assays in the 5637 cells.

HOXB7 overexpression was achieved using the CV702 plasmid (Shanghai GenePharma, Co., Ltd.). T24 cells were cultured in a 6-well plate and transfected with 5 μg HOXB7 overexpression plasmid or empty vector using Lipofectamine 3000 when confluence reached 70-90%. After 48 h of transfection, reverse transcription-quantitative (RT-q)-PCR and western blot analyses were performed to evaluate transfection efficiency. Given the differential expression of HOXB7 between 5637 and T24 cells, HOXB7 expression was knocked down in 5637 cells (which exhibit high baseline HOXB7 expression) and overexpressed HOXB7 in T24 cells (which exhibit low baseline HOXB7 expression) to investigate its functional role. A similar approach was previously used to study the functional effects in BC cells (14).

### Lentiviral vector construction

Lentiviral vectors encoding a short hairpin RNA (shRNA) for the knockdown of HOXB7 and a scramble shRNA control were provided by Shanghai GenePharma Co., Ltd. The transfection process followed the established protocols. To establish a stably transfected cell line, the transfected cells were cultured in media supplemented with 5 µg/ml puromycin. RT-qPCR was used to assess the mRNA expression levels 72 hours after infection.

### RT-qPCR

Total RNA was extracted using TRIzol^®^, according to the manufacturer’s protocol. RNA (2µg) was reverse-transcribed into cDNAs using a Hifair^®^ III First Strand cDNA Synthesis Kit (cat. no. 1114ES10; Shanghai Yeasen Biotechnology Co., Ltd.). For reverse transcription, the temperature protocol was 25°C for 5 min, 55 °C for 15 min, and 85°C for 5 min. qPCR was conducted using primers synthesized by Sangon Biotech Co., Ltd. The primer sequences for qPCR were as follows:

GAPDH forward, 5’-CAGGAGGCATTGCTGATGAT-3’ and reverse, 5’-GAAGGCTGGGGCTCATTT-3’; and HOXB7 forward, 5’-CGAGAGTAACTTCCGGATCTAC-3’ and reverse, 5’-GGTCTTGTTCTCCTTTTTCCAC-3’.

The qPCR thermocycling conditions were 40 cycles of 25°C for 5 sec, 55°C for 15 sec, and 85°C for 5 sec. Each sample was measured in triplicate to ensure reliability. Gene expression levels in the cells were normalized relative to GAPDH expression using the 2^−ΔΔCq^ method.

### CCK8 assay

A total of 4x10^3^ transfected cells were seeded into each well of a 96-well plate. Cell viability was measured on days 0, 1, 2 and 3 after transfection. For each time point, 10 μl CCK8 solution was added to each well, and cells were incubated for a further 2-h. Then, the absorbance was measured at 450 nm using a microplate reader (Synergy H1, Omega Bio-Tek, Inc.).

### EdU staining assay

The Cell-Light™ EdU Apollo643 *In Vitro* Imaging Kit (cat. no. C10310-2, Guangzhou RiboBio Co., Ltd.) was used to evaluate cell proliferation according to the manufacturer’s instructions. Briefly, BC cells (3x10^3^) were seeded into each well of a 96-well plate and allowed to adhere for 24 h. Subsequently cells were cultured with1.0 mg/ml EdU solution for 2 h to label the DNA of proliferating cells. The cells were fixed with 50 μl of 4% paraformaldehyde for 30 min. After fixation, the cells were treated with 0.5% Triton X-00 (cat. no. T8200; Beijing, Solarbio Science & Technology Co., Ltd.) for 10 min. Next, 100 μl Apollo staining solution was added to each well, and the plates were incubated in the dark for 30 minutes at room temperature. DNA staining was performed using Hoechst33342 to visualize the cell nuclei. Cell proliferation was assessed by observing the fluorescence of the stained cells under fluorescence microscopy (IX71, Olympus Corporation).

### Wound healing assay

A linear wound was created in a cell monolayer using a 1-ml sterile plastic pipette tip when the transfected cells had reached ~95% confluence in a 6-well plate. The cells were cultured in a media containing 2% FBS. Images of the wound area were captured using a microscope (BX53, Olympus Corporation) at 0, 24, and 48 h after wounding to monitor the rate of cell migration into the wound space.

### Colony forming assay

Transfected cells (500 cells/well) were seeded into a 6-well plate and complete media was added to each well to a final volume of 2 ml. The cells were cultured in a cell incubator, and the medium was refreshed every 3 days to maintain optimal growth conditions. After 2 weeks, the cells were fixed with 4% paraformaldehyde and stained with 0.1% crystal violet. Visible colonies were counted and imaged to assess the efficiency of the transfection and the proliferative capacity of the cells.

### Transwell assay

For the migration assay, 2x10^4^ treated and control BC cells were added to the upper chamber of a 24-well Transwell (8.0-mm, NEST Biotechnology, Wuxi, China) in 200 μl serum-free culture medium. The lower chamber was filled with 600 μl complete media.

For the invasion assay, Matrigel (cat. no. 356234, Corning Inc) was used to precoat the upper chamber for 24 h. Subsequently, 4x10^4^ treated and control BC cells were seeded into the upper chamber in 200 μl serum-free culture medium. The lower chamber was filled with 600 μl of 10% FBS complete culture medium.

After 24 or 48 h of incubation, the cells on the upper layers of upper chamber membranes were gently removed using a cotton swab. The cells that had migrated or invaded the lower surface of the membrane were fixed with 4% paraformaldehyde at room temperature for 30 min, followed by staining with 0.1% crystal violet for 30 min at room temperature. The number of cells was quantified in four randomly selected fields of view using a light microscope at x100 magnification.

### Flow cytometry assay for detecting cell apoptosis

Cells were harvested and resuspended in 500 µl binding buffer. Subsequently, the cells were stained using an Annexin V-FITC/PI Apoptosis kit according to the manufacturer’s protocol (cat. no. KGA108, Nanjing KeyGen Biotech Co., Ltd.). Staining was performed in the dark for 15 min. After staining, the percentage of apoptotic cells was determined using a BD FACS Canto II flow cytometer (BD Biosciences.).

### Western blotting assay

Cells were lysed in RIPA lysis buffer with protease and phosphatase inhibitors (cat. no. P1045, Beyotime Institute of Biotechnology). Protein concentrations were measured using a BCA protein assay kit (cat. no. P0010, Beyotime Institute of Biotechnology). Equal quantities of protein were mixed with SDS buffer, denatured, and prepared for gel electrophoresis. Sample were resolved on 6-15% SDS gels and resolved using SDS-PAGE (cat. on. P0012A, Beyotime Institute of Biotechnology) and transferred to a PVDF membrane (cat. on. 0021, MilliporeSigma). Membrane were blocked using 5% skimmed milk and incubated overnight at 4˚C with the following primary antibodies: HOXB7 (cat. on. 12613-1-AP; 1:1000; ProteinTech Group, Inc.), Vimentin (cat. on. 10366-1-AP; 1:2000; ProteinTech Group, Inc.), E-cadherin (cat. on. 20874-1-AP; 1:1000; ProteinTech Group, Inc.), N-Cadherin (cat. on. 22018-1-AP; 1:1000; ProteinTech Group, Inc.), H-Ras (cat. on. 18295-1-AP; 1:1000; ProteinTech Group, Inc.), Raf-1(cat. on. 347271; 1:1000; ZenBio), P38 (cat. on. A14401; 1:1000; ABclonal), p-P38(cat. on. AP0526; 1:2000; ABclonal), JNK1/2 (cat. on. A18287; 1:500; ABclonal), p-JNK1/2 (cat. on. AP0473; 1:1000; ABclonal), ERK (cat. on. A16686; 1:1000; ABclonal), p-ERK (cat. on. AP0472; 1:1000; ABclonal), Bax (cat. on. 50599-2-lg; 1:2000; ProteinTech Group, Inc.), Bcl2 (cat. on. 26593-1-AP; 1:1000; ProteinTech Group, Inc.), MEK (cat. on. 11049-1-AP; 1:5000; ProteinTech Group, Inc.). p-MEK (cat. on. 310050; 1:1000; Zenbio) and β-actin (cat. on. 81115-1-RR; 1:500; ProteinTech Group, Inc.). After incubation, the membranes were washed with TBST and incubated with appropriate secondary antibodies (cat. on. PMK-014-097M/PMK-014-097S, HRP-conjugated goat anti-rabbit IgG, H+L, Bioprimacy, 1:5000). Following three washes with TBST, the target protein bands were detected using a high-sensitivity ECL chemiluminescence kit (cat. on. P2200, NCM Biotech).

### Xenograft mouse model

A total of ten 6-week-old NSG mice were purchased from Shanghai Model Organisms Center, Inc. (Shanghai, China) and maintained under specific pathogen-free (SPF) conditions. The mice were randomly divided into two groups (n=5 per group). Each mouse in the scramble control group was subcutaneously injected with 1x10^7^ lentivirus-infected scramble 5637 cells, while each mouse in the shHOXB7 group was injected with 1x10^7^ lentivirus-infected shHOXB7–5637 cells. All injections were performed in the right axilla using 100 μl PBS as the suspension medium. Tumor growth was monitored by measuring the tumor length and width every 3 days, and tumor weights were recorded at the end of the study. Tumor volume was calculated using the formula: volume = length x width^2^/2 at the end of the 7-week experimental period. The maximum tumor diameter permitted in this study was 15 mm, in accordance with ethical guidelines. The mice were euthanized by cervical dislocation, and the tumors were harvested for weight measurement, immunohistochemical staining, and western blot analysis. The animal experiment was approved by the Animal Research Ethics Committee of Guizhou Medical University (approval no. 2201615) and performed in accordance with the Guidelines for Animal Experiments of Laboratory Animals.

### Statistical analysis

Data are presented as the mean ± standard deviation (SD) of at least three independent experiments. Differences between the two groups were determined using an unpaired Student’s *t*-test. The correlations between the clinical characteristics in the BC TMA were analyzed using a chi-square (χ^2^) test. ANOVA was used to compare differences between three or more groups. A Levene’s test was used to assess the homogeneity of variances, followed by either a Tukey’s *post-hoc* test (for equal variances) or the Games-Howell test (for unequal variances), depending on the uniformity of variance. All statistical analyses were performed using GraphPad version 8.0 (GraphPad Software Inc.). A two-sided *P* < 0.05 was considered to indicate a statistically significant difference.

## Results

### HOXB7 expression is upregulated in BC tissues

**Figure 1:**
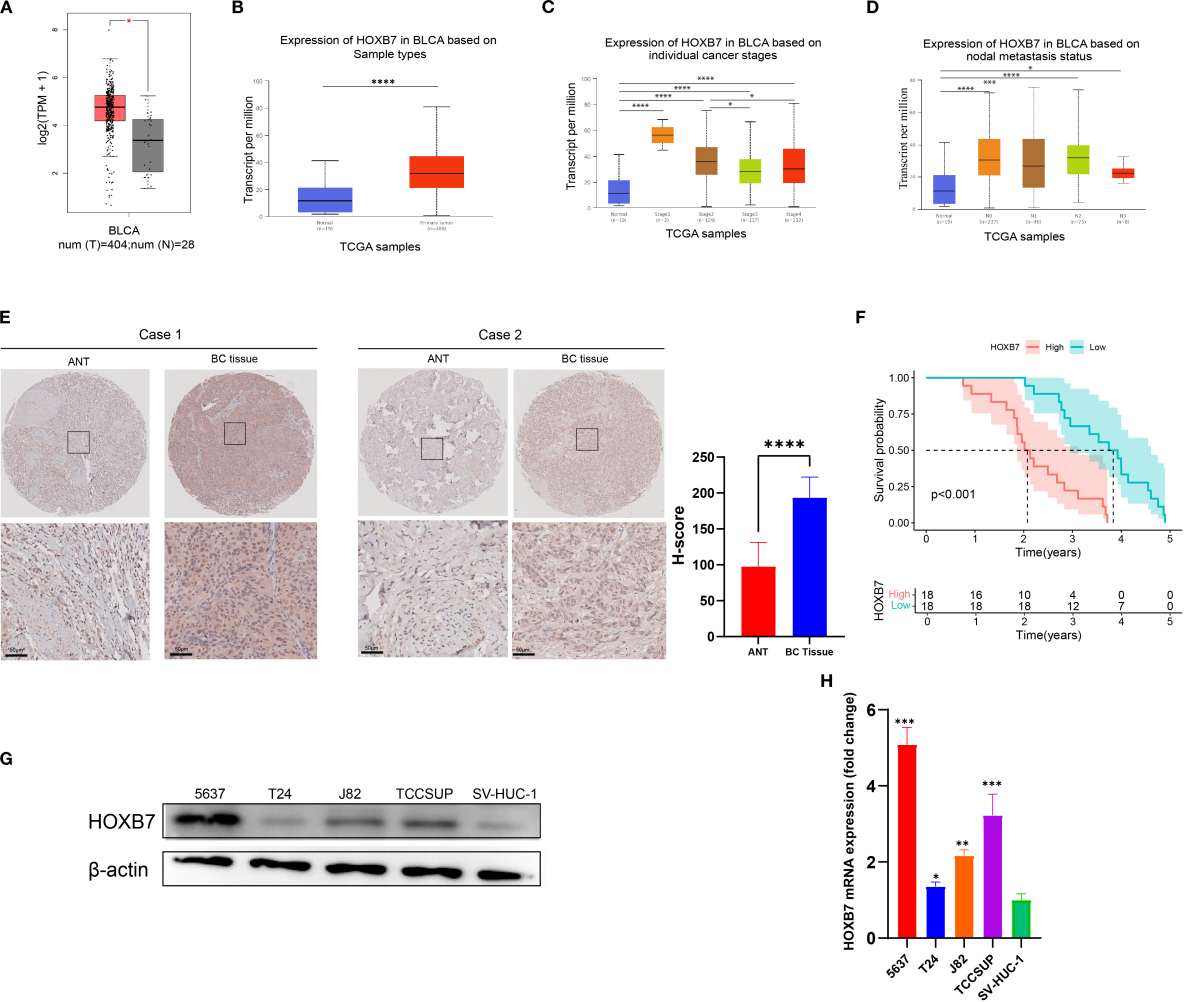
HOXB7 is upregulated in BC and correlates with poor prognosis. **(A)** HOXB7 mRNA expression in normal and tumor tissues was analyzed using the GEPIA database (TCGA normal + GTEx data). **(B–D)** UALCAN database analysis showing HOXB7 expression levels in BLCA stratified by **(B)** tissue type (normal vs. primary tumor), **(C)** individual pathological stages, and **(D)** lymph node metastasis status. **(E)** Representative immunohistochemical (IHC) staining of HOXB7 in paired ANT and BC tissues with quantification of H-scores. **(F)** Survival curves based on follow-up data from 36 BC patients show that high HOXB7 expression is significantly associated with worse overall survival. **(G, H)** HOXB7 expression was evaluated in BC cell lines (5637, T24, J82, TCCSUP) and SV-HUC-1 by western blot **(G)** and RT-qPCR **(H)**. Data are presented as mean ± SD. Statistical significance: *P < 0.05; **P < 0.01; ***P < 0.001; ****P < 0.0001.

To evaluate the expression pattern of HOXB7 in BC, the GEPIA database was first used to analyze data from TCGA and GTEx, revealing that HOXB7 expression was significantly higher in BC tumor tissues compared with normal bladder tissues (**Figure 1A**). Consistently, UALCAN analysis based on TCGA datasets confirmed the elevated expression of HOXB7 in primary BC tissues relative to normal samples (**Figure 1B**). Further stratification by tumor stage and lymph node metastasis status demonstrated that HOXB7 expression was significantly correlated with tumor progression, showing increased levels in advanced stages and in patients with lymph node involvement (**Figures 1C, D**). To validate these findings at the protein level, immunohistochemical (IHC) staining was performed on 36 paired BC and adjacent normal tissue (ANT) specimens. The results demonstrated markedly higher HOXB7 expression in BC tissues compared with matched normal counterparts (**Figure 1E**). Quantification of IHC staining confirmed this difference to be statistically significant (p < 0.0001). Survival analysis based on clinical follow-up data from these 36 patients revealed that high HOXB7 expression was significantly associated with poorer overall survival (p < 0.001; **Figure 1F**). Moreover, upregulated expression of HOXB7 was significantly associated with pathological grade and tumor stage (**Table 1**). Additionally, we assessed HOXB7 expression in four human BC cell lines (5637, T24, J82, and TCCSUP) and in the immortalized normal urothelial cell line SV-HUC-1. Both western blot and RT-qPCR analyses revealed elevated HOXB7 expression in BC cell lines, with the highest expression observed in 5637 cells and the lowest in T24 cells (**Figures 1G, H**). Based on these results, 5637 and T24 cell lines were selected for subsequent functional assays.

**Table 1:** Relationship between HOXB7 expression and clinicopathological characteristics in the patients with BC.

Parameters	All n=36	HOXB7 expression		P-value
		Low, n=15	High, n=21	
Age(year)				0.3643
<66	16	8	8	
≥66	20	7	13	
Sex				0.2187
Male	34	15	19	
Female	2	0	2	
Pathological grade				<0.0001
Low	14	12	2	
High	22	3	19	
Tumor stage				
T1/T2	17	12	5	0.0009
T3/T4	19	3	16	

### HOXB7 knockdown inhibits the proliferation of 5637 cells and overexpression promotes T24 cell proliferation

**Figure 2:**
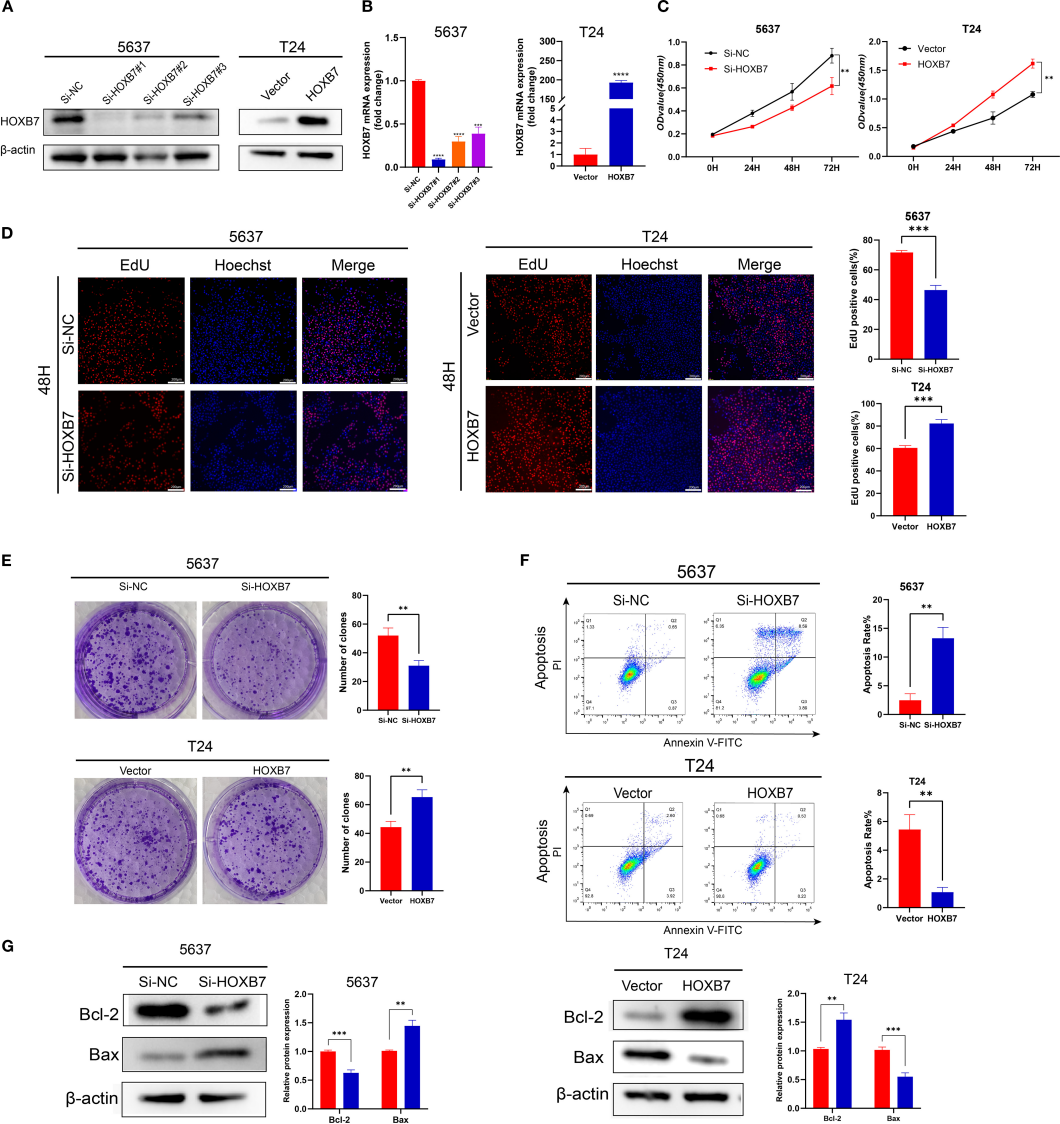
HOXB7 promotes BC cell proliferation and inhibits apoptosis. **(A, B)** HOXB7 expression was silenced in 5637 cells using three independent siRNAs and overexpressed in T24 cells using a HOXB7 expression plasmid. Knockdown and overexpression efficiency were confirmed by western blot and RT-qPCR. **(C)** CCK-8 assays were performed to evaluate the effects of HOXB7 on cell proliferation in 5637 and T24 cells. **(D)** EdU assays further demonstrated that HOXB7 knockdown reduced proliferation in 5637 cells, while HOXB7 overexpression enhanced proliferation in T24 cells. **(E)** Colony formation assays showed that HOXB7 knockdown decreased, while HOXB7 overexpression increased, the clonogenic capacity of BC cells. **(F)** Flow cytometry analysis revealed that HOXB7 knockdown promoted apoptosis in 5637 cells, whereas overexpression inhibited apoptosis in T24 cells. **(G)** Western blot analysis of apoptosis-related proteins showed that HOXB7 knockdown upregulated Bax and downregulated Bcl-2 in 5637 cells, while HOXB7 overexpression had the opposite effect in T24 cells. Data are presented as mean ± SD. **P < 0.01; ***P < 0.001; ****P < 0.0001.

To modulate HOXB7 expression in BC cells, siRNA-mediated knockdown was performed in 5637 cells, while HOXB7 was ectopically overexpressed in T24 cells using a pcDNA3.1-HOXB7 plasmid. Western blotting and RT-qPCR confirmed efficient knockdown and overexpression of HOXB7, respectively (**Figures 2A, B**). The effect of HOXB7 on cell proliferation was assessed using CCK-8 and EdU assays. Silencing HOXB7 significantly suppressed the proliferation of 5637 cells, while HOXB7 overexpression markedly enhanced the proliferation of T24 cells (**Figures 2C, D**). Consistent results were obtained in colony formation assays, where HOXB7 knockdown reduced the number of colonies formed in 5637 cells, and HOXB7 overexpression increased clonogenic growth in T24 cells (**Figure 2E**). To investigate whether the observed proliferative changes were associated with apoptosis, flow cytometry analysis was performed. HOXB7 knockdown significantly increased the apoptosis rate in 5637 cells, whereas HOXB7 overexpression suppressed apoptosis in T24 cells (**Figure 2F**). Further analysis of apoptosis-related protein expression revealed that HOXB7 silencing downregulated anti-apoptotic Bcl-2 and upregulated pro-apoptotic Bax in 5637 cells. Conversely, in T24 cells, HOXB7 overexpression led to increased Bcl-2 and decreased Bax levels (**Figure 2G**).

### HOXB7 promotes migration and invasion in BC cell lines and modulates the expression of EMT markers

**Figure 3:**
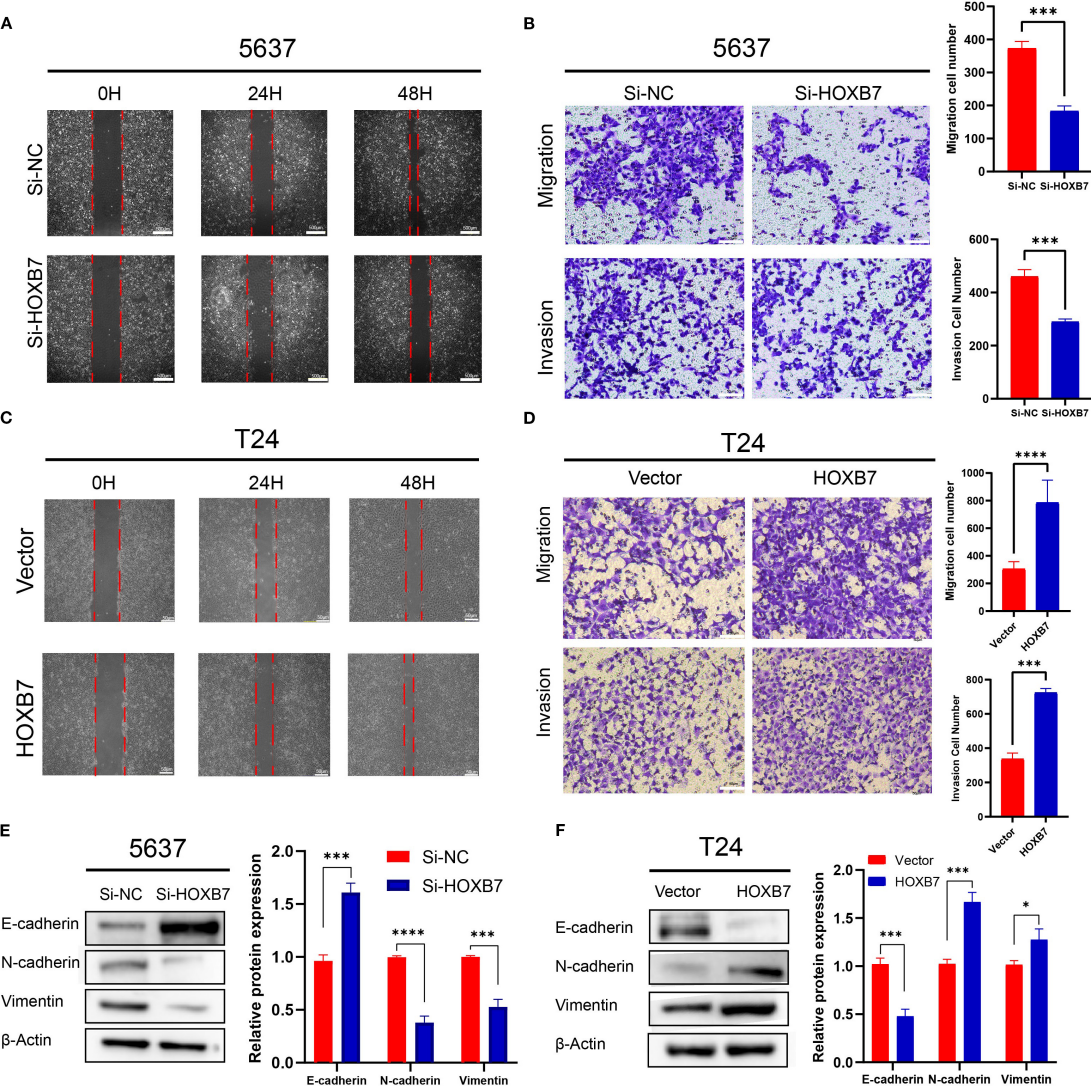
HOXB7 promotes EMT and enhances migration and invasion in BC cells. **(A, C)** Wound healing assays showed that HOXB7 knockdown inhibited migration in 5637 cells, while HOXB7 overexpression promoted migration in T24 cells. **(B, D)** Transwell migration and invasion assays demonstrated that silencing HOXB7 significantly reduced both migratory and invasive capacities in 5637 cells, whereas HOXB7 overexpression enhanced these capacities in T24 cells. **(E, F)** Western blot analysis revealed that HOXB7 knockdown upregulated the epithelial marker E-cadherin and downregulated the mesenchymal markers N-cadherin and Vimentin in 5637 cells. Conversely, HOXB7 overexpression in T24 cells led to decreased E-cadherin and increased N-cadherin and Vimentin levels. Data are presented as mean ± SD. *P < 0.05; ***P < 0.001; ****P < 0.0001.

EMT is a key process involved in cancer metastasis. To evaluate the role of HOXB7 in BC cell migration, wound healing assays were performed. Knockdown of HOXB7 significantly reduced the migratory ability of 5637 cells, while overexpression of HOXB7 enhanced migration in T24 cells (**Figures 3A, C**). Consistently, Transwell assays demonstrated that HOXB7 silencing significantly inhibited both migration and invasion of 5637 cells, whereas HOXB7 overexpression in T24 cells promoted these abilities (**Figures 3B, D**). To further explore whether HOXB7 affects EMT in BC cells, the expression levels of EMT-related markers were examined by western blot. In 5637 cells, HOXB7 knockdown led to increased expression of the E-cadherin and decreased levels of N-cadherin and Vimentin. Conversely, HOXB7 overexpression in T24 cells suppressed E-cadherin while upregulating N-cadherin and Vimentin (**Figures 3E, F**). These results indicate that HOXB7 facilitates the malignant progression of BC cells, at least in part, by promoting EMT.

### HOXB7 increases MAPK/MEK/ERK signaling

**Figure 4:**
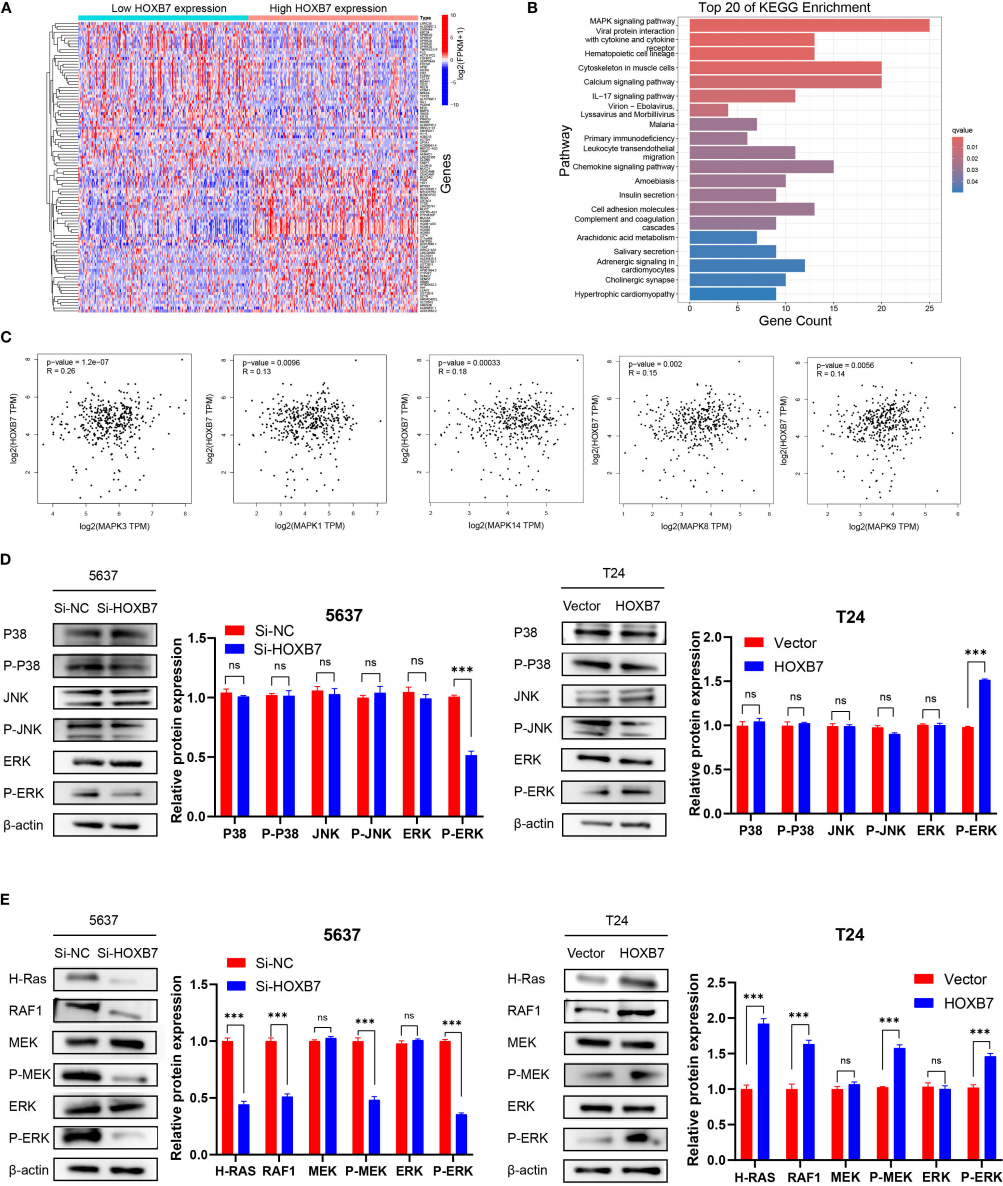
HOXB7 regulates the MAPK signaling pathway in BC. **(A)** Heatmap showing DEGs between high and low HOXB7 expression groups in BC based on TCGA transcriptome data. **(B)** KEGG pathway enrichment analysis of DEGs revealed significant involvement of the MAPK signaling pathway. **(C)** Correlation analysis using the GEPIA database showed that HOXB7 expression was positively associated with multiple MAPK pathway components, including MAPK3 (ERK1), MAPK1 (ERK2), MAPK14 (p38), MAPK8 (JNK1), and MAPK9 (JNK2). **(D)** Western blot analysis confirmed that HOXB7 modulates MAPK pathway activation in BC cell lines. HOXB7 knockdown in 5637 cells significantly reduced the phosphorylation of ERK1/2, whereas HOXB7 overexpression in T24 cells increased p-ERK levels. Total protein levels of ERK, JNK, and p38, and their phosphorylated forms were also assessed. **(E)** Western blot analysis of H-Ras/RAF1/MEK/ERK signaling in HOXB7-silenced 5637 cells and HOXB7-overexpressing T24 cells. Knockdown of HOXB7 decreased the expression of H-Ras, RAF1, p-MEK, and p-ERK, while overexpression of HOXB7 had the opposite effect. β-actin served as the loading control. Data are presented as mean ± SD. NS, Not Significant; ***P < 0.001.

To elucidate the regulatory mechanisms of HOXB7 in the occurrence and development of BC, patients with BC were stratified into high- and low- HOXB7 expression groups using data from the TCGA. The analysis revealed a significant difference in gene expression patterns between the two groups (**Figure 4A**). KEGG pathway analysis indicated that the DEGs influenced by HOXB7 were predominantly involved in the MAPK signaling pathway (**Figure 4B**). To further explore this relationship, key targets within the MAPK signaling pathway were identified and their correlation with HOXB7 expression in BC was validated using the GEPIA database. The analysis revealed a positive correlation between HOXB7 and key components of the MAPK pathway, including MAPK3, MAPK1, MAPK14, MAPK8, and MAPK9 (**Figure 4C**). Functionally, these components are known as ERK1, ERK2, P38, JNK1, and JNK2, respectively (**Figure 4C**). The results from the western blot analysis further confirmed that HOXB7 knockdown resulted in a significant reduction in the phosphorylation levels of ERK1/2, while total ERK levels remained unchanged in 5637 cells. No significant alterations were observed in the phosphorylation or total expression of P38 and JNK1/2. Conversely, in T24 cells, HOXB7 overexpression markedly increased the phosphorylation levels of ERK1/2 without affecting total ERK levels, whereas P38 and JNK1/2 levels, both phosphorylated and total, showed no significant changes (**Figure 4D**). These findings confirm that HOXB7 specifically regulates the activation of ERK1/2 within the MAPK signaling pathway in both 5637 and T24 cells, further emphasizing its pivotal role in modulating this pathway’s activity. In addition, cell function assays indicated that HOXB7 promoted the EMT phenotype of BC cells. Previous research established the importance of the Ras-mediated Raf/MEK/ERK pathway in EMT regulation. These findings support the hypothesis that HOXB7 may regulate the occurrence and development of BC by modulating the MAPK/MEK/ERK signaling pathway. Nevertheless, the crucial role of HOXB7 and the MAPK/MEK/ERK pathways in BC development has not been extensively reported and requires additional experimental validation. Thus, the changes in the H-Ras/Raf-1/MEK/ERK pathway following HOXB7 downregulation and overexpression were investigated, including H-Ras, Raf-1, MEK, p-MEK, ERK, and p-ERK. In 5637 cells, HOXB7 downregulation reduced the phosphorylation levels of MEK and ERK and decreased the expression of H-Ras and Raf-1. In contrast, HOXB7 overexpression significantly increased the expression of H-Ras, Raf-1, p-MEK, and p-ERK in T24 cells (**Figure 4E**).

### Confirmation of the role of MAPK/MEK/ERK signaling in the HOXB7-mediated effects

**Figure 5:**
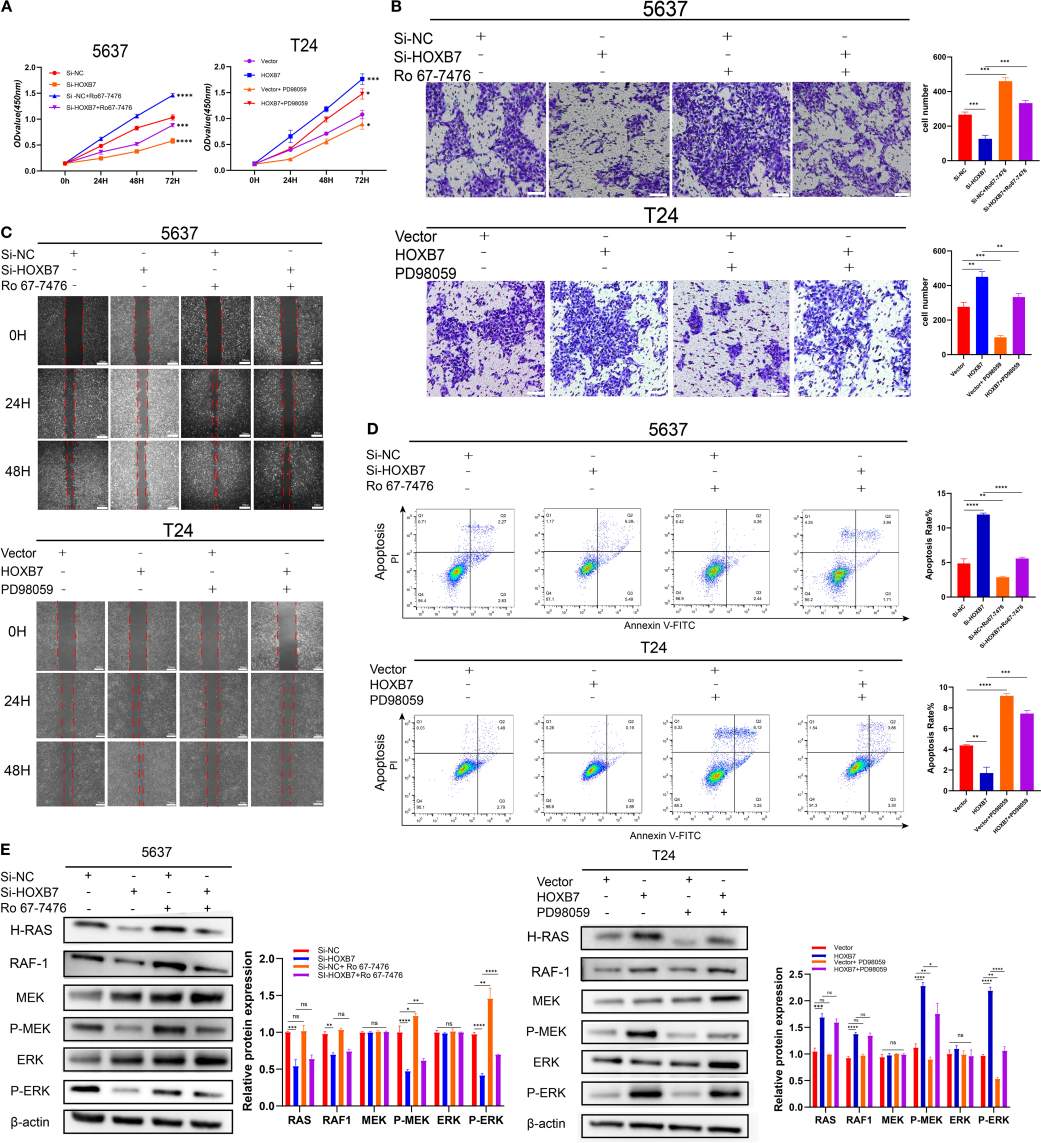
HOXB7 promotes BC progression via activation of the H-Ras/RAF1/MEK/ERK signaling pathway. **(A)** CCK-8 assays were used to assess the effects of the ERK pathway activator Ro67-7476 (1 μM) and the MEK inhibitor PD98059 (10 μM) on cell proliferation in HOXB7-knockdown 5637 cells and HOXB7-overexpressing T24 cells. **(B)** Transwell invasion assays demonstrated that Ro67–7476 restored the invasive capacity of HOXB7-silenced 5637 cells, while PD98059 inhibited the enhanced invasion induced by HOXB7 overexpression in T24 cells. **(C)** Wound healing assays showed that the migration-inhibitory effect of HOXB7 knockdown was partially reversed by Ro67-7476, and the migration-promoting effect of HOXB7 overexpression was suppressed by PD98059. **(D)** Flow cytometry analysis revealed that Ro67–7476 partially rescued the increased apoptosis caused by HOXB7 knockdown, while PD98059 restored apoptosis in HOXB7-overexpressing T24 cells. **(E)** Western blot analysis showed that Ro67–7476 reversed the decrease in MEK and ERK phosphorylation caused by HOXB7 knockdown, whereas PD98059 inhibited MEK and ERK activation induced by HOXB7 overexpression. Data are presented as mean ± SD. NS, Not Significant; *P < 0.05; **P < 0.01; ***P < 0.001; **** P < 0.0001.

To investigate whether the Ras/ERK signaling pathway mediates the functional effects of HOXB7 in BC cells, the ERK1/2-specific activator Ro67–7476 and the MEK inhibitor PD98059 were used for rescue experiments in 5637 and T24 cells. CCK-8 assays demonstrated that the proliferation-inhibitory effect of HOXB7 knockdown in 5637 cells was partially reversed by Ro67–7476 treatment (1 μM, 24 h), while the proliferation-enhancing effect of HOXB7 overexpression in T24 cells was suppressed by PD98059 (10 μM, 24 h) (**Figure 5A**). Similarly, Transwell assays showed that Ro67–7476 significantly restored the invasive capacity of HOXB7-silenced 5637 cells, whereas PD98059 attenuated the invasion-promoting effect of HOXB7 overexpression in T24 cells (**Figure 5B**). Wound healing assays further confirmed that the impaired migration of 5637 cells upon HOXB7 silencing was rescued by Ro67-7476, while PD98059 suppressed the enhanced migration caused by HOXB7 overexpression in T24 cells (**Figure 5C**). Flow cytometry analysis revealed that HOXB7 knockdown significantly increased apoptosis in 5637 cells, which was partially reversed by Ro67-7476. In T24 cells, HOXB7 overexpression reduced apoptosis, whereas PD98059 treatment increased the apoptotic rate, particularly in HOXB7-overexpressing cells (**Figure 5D**). Western blot analysis showed that HOXB7 knockdown decreased MEK and ERK phosphorylation in 5637 cells, and this effect was rescued by Ro67-7476. Conversely, HOXB7 overexpression enhanced phosphorylation of MEK and ERK in T24 cells, which was effectively suppressed by PD98059 (**Figure 5E**). These findings indicate that the Ras/MEK/ERK signaling pathway plays a critical role in mediating the tumor-promoting effects of HOXB7 in BC cells.

### HOXB7 facilitates tumorigenesis *in vivo*

**Figure 6:**
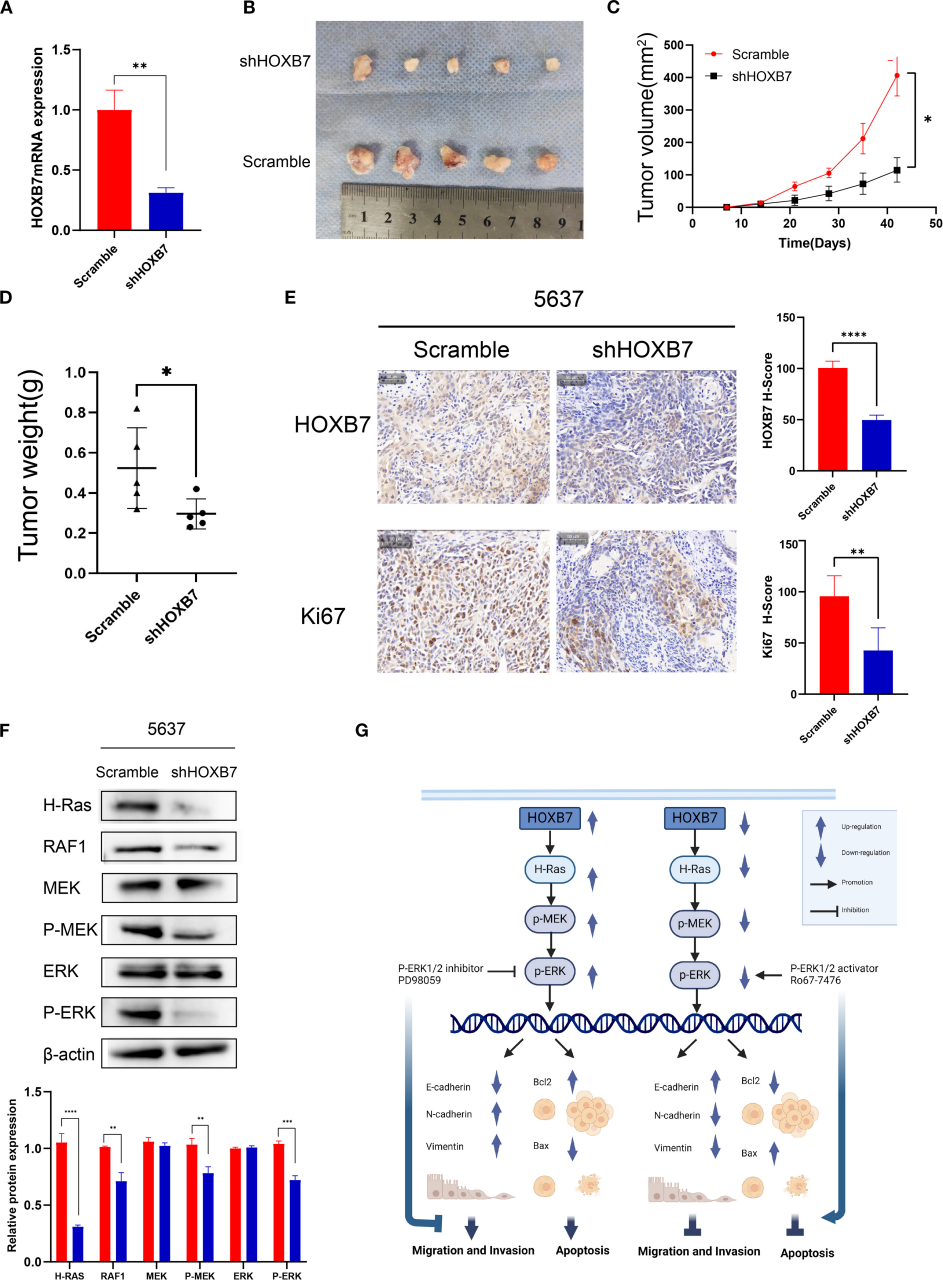
HOXB7 knockdown suppresses tumorigenesis *in vivo* via inhibition of the H-Ras/RAF1/MEK/ERK signaling pathway. **(A)** HOXB7 mRNA expression in stably transfected 5637 cells was confirmed by RT-qPCR. **(B)** Representative images of xenograft tumors formed in nude mice injected with 5637 scramble or shHOXB7 cells (n = 5 per group). **(C, D)** Tumor volume was monitored over time, and tumor weight was measured at the endpoint. Both were significantly reduced in the shHOXB7 group compared to control. **(E)** Immunohistochemical (IHC) staining of xenograft tissues showed reduced HOXB7 and Ki-67 expression in the shHOXB7 group, with corresponding decreases in H-score values. **(F)** Western blot analysis demonstrated that HOXB7 knockdown suppressed the expression of H-Ras and RAF1, as well as phosphorylation of MEK and ERK in xenograft tumors. β-actin was used as a loading control. **(G)** Schematic diagram summarizing the proposed mechanism: HOXB7 promotes BC cell proliferation, migration, and EMT while inhibiting apoptosis through activation of the H-Ras/MEK/ERK signaling pathway. Data are presented as mean ± SD. *P < 0.05; **P < 0.01; ***P < 0.001; ****P < 0.0001.

To investigate the *in vivo* role of HOXB7 in tumor development, a subcutaneous xenograft model was established using 5637 BC cells stably transfected with either shHOXB7 or scramble control. RT-qPCR confirmed efficient knockdown of HOXB7 expression (**Figure 6A**). Tumors derived from shHOXB7-transfected cells were significantly smaller in both volume and weight compared with those in the control group (**Figures 6B–D**). Immunohistochemical staining revealed a marked reduction in HOXB7 and Ki-67 expression in the shHOXB7 group, indicating decreased proliferative activity (**Figure 6E**). Western blot analysis of tumor tissues showed that HOXB7 knockdown led to downregulation and decreased phosphorylation of several key components in the H-Ras/MEK/ERK signaling pathway, including H-Ras, RAF1, p-MEK, and p-ERK (**Figure 6F**). A schematic diagram illustrating the proposed mechanism is shown in **Figure 6G**.

## Discussion

Recurrence and progression remain major challenges in treating BC (15). Approximately half of the patients with MIBC experience local recurrence or systemic metastasis after surgery. Therefore, it is crucial to explore novel therapeutic targets to reduce recurrence and metastasis and improve the prognosis of patients with BC.

As a key member of the HOX gene family, HOXB7 expression is upregulated in various malignant tumors and is associated with advanced-stage cancer, metastasis, tumor proliferation, and poor patient survival. HOXB7 upregulation has been observed in hepatocellular carcinoma (16), oral squamous cell carcinoma (17), colorectal cancer (18), breast cancer (19), and intrahepatic cholangiocarcinoma (20). Concurrently, the present bioinformatics analysis showed that HOXB7 expression was elevated in BC compared with normal tissue, a finding corroborated by the TMA immunohistochemistry, which showed HOXB7 upregulation in BC tissues relative to ANT and a positive correlation with pathological grade and TNM stage. HOXB7 knockdown suppressed BC cell proliferation in CCK8, EdU, immunofluorescence, and colony formation assays, whereas overexpression produced the opposite effects. In xenograft tumor experiments, HOXB7 knockdown significantly reduced tumor growth and Ki67 expression These results support an oncogenic role for HOXB7 in BC cell proliferation. A similar study showed that HOXB7 was highly expressed in hepatocellular carcinoma, where it promoted cell proliferation, upregulated cancer stem cell markers EPCAM and NANOG, enhanced c-Myc and Slug expression, and promoted tumor growth (16). Furthermore, HOXB7 was identified as a pivotal factor in increasing basic fibroblast growth factor (bFGF) secretion within the tumor environment and in promoting the proliferation and differentiation of bone marrow mesenchymal progenitors (21). ​ Collectively, HOXB7 overexpression appeared to play a central role in the initiation and progression of BC. EMT is a biological characteristic of malignant tumors. In the present study, HOXB7 was shown to enhance the mesenchymal phenotype based on the increased expression of N-cadherin and Vimentin and reduced expression of E-cadherin. Conversely, HOXB7 knockdown elicited the opposite effect. Similarly, previous studies reported the EMT-promoting role of HOXB7 in tumors. In gastric cancer, HOXB7 disrupted the F-actin structure and promoted the EMT (22). Furthermore, HOXB7 facilitated the EMT and stemness of hepatoma cells through the PI3K/AKT/Slug signaling pathway (16). HOXB7 upregulates bFGF, supporting the activation of the Ras/Rho pathway, which induces EMT and promotes the acquisition of aggressive properties such as tumorigenicity, migration, and invasion (23). HOXB7 overexpression induced TGFβ2 expression, thereby enhancing cell motility and invasiveness (24). Therefore, HOXB7 may drive distinct migratory properties and promote cell fate plasticity by activating EMT, thus facilitating metastasis and tumor recurrence in BC.

The RAS protein family, known for its role in cell signaling, can influence cell death, proliferation, and metabolic stress responses through the Raf-1/MEK/ERK and Rac1/MKK7/JNK pathways, which in turn modulate tumor progression (25). In the present, investigation into the role of HOXB7 in BC cells, bioinformatics analysis revealed that HOXB7 expression was associated with the MAPK signaling pathway in BC. Moreover, the functional experiments, including loss-of-function and gain-of-function approaches, underscored the pivotal role of the Ras-ERK signaling pathway in governing cell proliferation, EMT, and apoptosis in BC cells.

HOXB7 has been shown to enhance the proliferation and metastasis of liver cancer cells by activating the MAPK/ERK pathway (26). Similarly, in pancreatic cancer, HOXB7 knockdown reduced ERK phosphorylation, affecting the Rho family of GTPases and consequently diminishing cell protrusions and invasiveness (27). In the present study, GEPIA database analysis and western blot data confirmed the interaction between HOXB7 and the MAPK/MEK/ERK signaling pathway in BC. ERK1/2, central to the MAPK pathway, can exert both pro- and anti-apoptotic effects depending on cellular context (28, 29). Here, HOXB7 knockdown enhanced apoptosis by inhibiting Ras-ERK activation, whereas HOXB7 overexpression suppressed apoptosis—effects modulated by ERK phosphorylation status. These results demonstrated that HOXB7 promotes BC cell proliferation and inhibits apoptosis via Ras-ERK–mediated regulation of Bax and Bcl-2.

Interestingly, additional research indicated that HOXB7 exhibited effects beyond its oncogenic role in tumors. Chen et al. (30) identified a dual role for HOXB7, which initially inhibited the development of breast tumors but later promoted tumor growth and spread to the lungs. Moreover, HOXB7 overexpression has been linked to macrophage recruitment and activation (24) as well as to the stimulation of DNA double-stranded break repair, thereby improving breast cancer survival after irradiation (31). It also promoted tumor immunity and sensitivity to immunotherapy. Whether HOXB7 has similar effects in BC requires further experimental verification and will form the focus of our future research.

The present study systematically demonstrated that HOXB7 promotes BC progression by enhancing cell proliferation and EMT, while inhibiting apoptosis through activation of the H-Ras/Raf-1/MEK/ERK signaling pathway, as validated in both *in vitro* and *in vivo* models. While this pathway is known to contribute to the progression of various malignancies, its involvement as a downstream effector of HOXB7 in BC has not been previously elucidated. Our findings fill a critical gap in the current understanding of BC biology and underscore the importance of the HOXB7–H-Ras/ERK axis as a potential target for therapeutic intervention.

From a translational perspective, our findings establish HOXB7 as both a prognostic biomarker and a therapeutic target in BC. HOXB7 can be targeted indirectly via MEK/ERK inhibition or more directly through HOX/PBX interaction inhibitors such as HXR9, which has shown preclinical anti-tumor efficacy in other HOX-driven cancers (32). As an upstream activator of ERK signaling, HOXB7 may define a molecular subgroup that could benefit from clinically available MEK/ERK inhibitors (e.g., trametinib, selumetinib), either alone or in combination with cisplatin-based chemotherapy. Beyond direct tumor growth inhibition, targeting the HOXB7–H-Ras/ERK axis may also modulate anti-tumor immunity. Constitutive PD-L1 expression in tumors is strongly dependent on MAPK pathway activation, with ERK 1/2 promoting PD-L1 transcription via c-JUN/AP-1 and enhancing PD-L1 mRNA stability. Preclinical studies have shown that ERK inhibition—especially when combined with JNK blockade—can reduce PD-L1 expression, potentially enhancing the efficacy of PD-L1 immune checkpoint blockade (33–35). Future studies should clarify whether HOXB7-driven ERK activation in BC contributes to immune evasion, thereby informing combination strategies that integrate HOXB7 or ERK inhibitors with immunotherapy. Collectively, these insights highlight the HOXB7–ERK axis as a promising target for precision oncology, offering potential for both prognostic stratification and personalized therapy.

This study has several limitations. First, the 5637–T24 cell line model was selected for their contrasting endogenous HOXB7 expression, enabling us to assess both gain- and loss-of-function within a well-established, literature-supported system for bladder cancer. While additional aggressive lines such as J82 or TCCSUP could offer complementary perspectives, our results were consistent across both lines, and this model has been widely used to represent distinct biological states of the disease. Second, our primary focus was to elucidate HOXB7’s biological functions and H-Ras/ERK–mediated mechanisms; although the tumor immune microenvironment was not examined, it remains an important future direction to clarify whether HOXB7-driven ERK activation contributes to immune modulation. Third, potential off-target effects are a well-recognized consideration in RNA interference experiments. we minimized such effects by using multiple independent, sequence-validated constructs, confirming knockdown at both mRNA and protein levels, and observing consistent phenotypic outcomes across assays. Finally, while larger independent patient cohorts were not available, our well-characterized cohort with complete follow-up provided statistically robust associations between HOXB7 expression and pathological parameters, supporting the reliability of our conclusions. Future multicenter studies with larger cohorts are warranted to confirm these findings.

In conclusion, this study provides comprehensive experimental evidence highlighting the pivotal role of HOXB7 in BC progression via modulation of the H-Ras/ERK pathway. These insights offer promising directions for the development of targeted therapies aimed at improving clinical outcomes for patients with BC and potentially other malignancies driven by similar molecular mechanisms.

## Data Availability

The original contributions presented in the study are included in the article/supplementary material. Further inquiries can be directed to the corresponding author.
